# Color as physical/conceptual attribute: Color word translation by Chinese postgraduate students

**DOI:** 10.1371/journal.pone.0342447

**Published:** 2026-02-10

**Authors:** Hui Wang, Guangsheng Lv, Xiaoming Tian

**Affiliations:** School of Humanities and Foreign Languages, Xi’an University of Posts and Telecommunications, Xi’an, Shaanxi, China; National University of Malaysia Faculty of Education: Universiti Kebangsaan Malaysia Fakulti Pendidikan, MALAYSIA

## Abstract

Color words present a persistent translation challenge because they function as both perceptual descriptors and carriers of conceptual meaning, and translators must decide which dimension to foreground across genres. Grounded in conceptual metaphor theory, this study investigates how 21 Chinese postgraduate students majoring in translation, many with strong technical backgrounds, translate color words in technical, promotional, and literary contexts, and what cognitive and cultural considerations drive their strategy choices. Participants, recruited from six universities in Shaanxi Province with strong technical profiles, were all experienced in translating technical texts. The findings reveal that students’ treatment of color words varies according to the text type: in technical texts, they predominantly draw on the metaphorical schema of COLOR AS PHYSICAL ATTRIBUTE, prioritizing visual precision and literal meaning. In contrast, in literary or promotional texts, they tend to activate the schema of COLOR AS CONCEPTUAL ATTRIBUTE, focusing on cultural and symbolic meanings. Their translation strategies, ranging from literal rendering to free translation and selective annotation, are shaped by the communicative function and symbolic significance of color words. This study demonstrates that color words serve both as perceptual phenomena and carriers of culturally embedded conceptual meanings, and that translators must actively navigate these dimensions to produce functional and culturally appropriate translations. These results contribute a schema-based explanation of genre-sensitive metaphor regulation in color-word translation and inform translator education by recommending differentiated training that balances precision with cultural resonance.

## Introduction

As technical specifications, product marketing, and media narratives circulate globally, small shifts in color terms can mislead users, weaken persuasion, or distort literary imagery. Color words, therefore, represent an urgent and practical translation issue because they simultaneously encode perceptual description and culture-specific symbolism. This challenge is especially pronounced when translating color words, which carry not only visual meanings but also deep symbolic and emotional connotations that vary across languages and cultures [1]. Understanding how these meanings are transferred in translation is crucial, particularly as global communication increasingly relies on accurate and culturally sensitive translations. Yet empirical research has rarely examined how novice translators with technical training regulate this duality across different text types, leaving unclear what cognitive mechanisms and strategy repertoires underpin their decisions.

Color constitutes a significant domain in human cognition, with language serving as the primary tool for encoding and conveying color. While the physical attributes of color are objectively represented on a spectrum, the linguistic terms used to describe color differ greatly across languages, influenced by cultural and psychological factors [2]. The relationship between color words and cognition has been widely studied, with research emerging since the mid-20th century that examines the interplay between language and perception [3]. However, much of this research has focused on color in general linguistic and cognitive terms, while the specific challenges of translating color words, particularly in specialized fields such as technical translation, remain underexplored. This research gap is critical, as color words often acquire specific cultural and psychological meanings that are vital to understanding and effectively translating texts across cultural boundaries.

In this study, color words are defined as linguistic expressions used to describe colors, which include both their basic visual characteristics and their extended cultural, psychological, and symbolic meanings. These words are examined within three primary dimensions: cultural, cognitive, and visual. The cultural dimension refers to the symbolic connotations embedded in social traditions, which may require translation strategies such as domestication or foreignization to balance fidelity and readability [4,5]. The cognitive dimension concerns the mental processes by which individuals perceive and conceptualize colors [2,5], while the visual dimension deals with the directly perceivable properties of color, such as hue and brightness, which are particularly important in technical documents where accuracy is crucial [6].

If the translation of color words is limited to their literal meanings without considering these deeper dimensions, communication can fail to capture the intended significance. For instance, the Chinese term “红眼病” (literally “red-eye disease”) is medically accurate as a translation of conjunctivitis, but culturally, it refers to “jealousy”. A literal translation, such as “He really has red-eye disease; he can’t bear to see others doing well,” would confuse English readers. The culturally equivalent phrase, “green with envy,” conveys the intended meaning. This example highlights the importance of addressing the cultural and psychological dimensions of color words, which are often overlooked in technical translation settings. To avoid such errors, translators must actively engage with these deeper meanings, requiring not just linguistic skill but also cultural and psychological awareness [7].

Against this backdrop, the present study explores how 21 Chinese postgraduate students majoring in translation approach the translation of color words in different contexts. These participants, recruited from six universities with strong technical profiles, provide a unique opportunity to examine how technical training influences the handling of culturally and psychologically loaded color words. Prior research indicates that translators working in technical domains often prioritize precision and functional clarity over cultural and emotional nuances [8].

To address this gap, the study uses focus group interviews to investigate the participants’ cognitive and cultural strategies for translating color words, exploring their awareness of cultural connotations, and the cognitive mechanisms influencing their decisions. This responds to Zou’s [9] call for integrating cultural awareness into translator education, encouraging students to make more informed, context-sensitive translation decisions. The study explores four key research questions: (I) Are students aware of cultural connotations associated with color words? (II) How do cognitive mechanisms shape their word choices in translation? (III) How do text types and functions shape their treatment of color words? (IV) What strategies do they employ for translating color words?

This study aims to make both theoretical and pedagogical contributions. Theoretically, it highlights how the symbolic and psychological aspects of language influence translation, enhancing understanding of color word translation in intercultural contexts. It supports Tursunovich’s assertion regarding the critical role of linguistic and cultural knowledge in ensuring translation quality [1]. Methodologically, the study employs qualitative data to explore the cognitive processes of technically trained translators, providing insights into the internal reasoning that shapes translation decisions. Pedagogically, the study offers guidance for curriculum design, emphasizing the importance of training translators to recognize the cultural, psychological, and symbolic significance of color words in their work.

## Literature review

Color words have drawn significant attention in translation studies. Color word translation goes beyond conveying visual attributes; it also encapsulates cultural backgrounds and psychological connotations, which profoundly influence translators’ choices [10]. Scholars agree that effective translation of color words should balance literal accuracy with symbolic resonance, highlighting the need for nuanced strategies tailored to the text type and target audience [11].

Across different contexts, the functional and symbolic roles of color diverge. In technical documents, translation tends to emphasize precision and functionality, with colors like red or blue used primarily for signaling or descriptive purposes [12,13]. In contrast, literary translations demand deeper engagement with cultural connotations and metaphorical meanings, as colors often serve as vehicles for emotional tone or symbolic narrative [14]. This distinction illustrates how context-specific constraints shape translators’ interpretive frameworks and influence whether physical or psychological nuances are prioritized.

These dynamics are equally relevant in audiovisual and advertising translation, where color plays a crucial role in audience perception and emotional engagement. Puchala [15] and Jonauskaite et al. [13] demonstrate how visual stimuli interact with psychological associations to affect meaning transfer, while Tursunovich [1] emphasizes translators’ tasks in reconciling these associations with cultural expectations. Rather than treating color words as static referents, recent scholarship frames them as cognitively and emotionally charged units, subject to variation across cultures and communicative contexts [16,17].

This symbolic complexity is further compounded by individual translators’ cognitive and cultural dispositions. Translation, as Hanada [18] and Elliot & Maier [19] argue, is not merely linguistic substitution but a form of cultural reconstruction shaped by the translator’s internal responses. Cognitive factors, such as familiarity with metaphorical systems, emotional reactions to colors, and awareness of cultural variation, profoundly impact word choices in translation [20]. Accordingly, strategies like free translation or cultural substitution become essential for mediating symbolic mismatches across linguistic and psychological domains [8,9].

Despite extensive research on color word translation, empirical research on how novice translators, particularly those from technical disciplines, engage with such symbolism remains scarce. Much of the existing scholarship either concentrates on single-context analyses or emphasizes theoretical models of metaphor and cultural variation, while giving less attention to how student translators operationalize these concepts in practice. This gap is especially salient in the case of science and engineering students, who may privilege precision over symbolic nuance when translating color words. Although psychological studies on color perception have deepened our understanding of how sensory stimuli transform into cognitive and emotional experiences [21], their implications for translation pedagogy remain underexplored. Consequently, there is a pressing need for empirical investigations that examine how disciplinary training intersects with translation strategies in handling color symbolism.

This study addresses these gaps by investigating 21 postgraduate translation students from science and engineering universities in China. It explores how their disciplinary backgrounds shape their engagement with psychological, cultural, and contextual symbolism in translating color words across literary, technical, and multimodal contexts. By doing so, it connects theoretical insights on color word translation with the practical realities faced by student translators navigating multiple textual and cultural expectations.

## Theoretical considerations

Conceptual metaphor theory, proposed by Lakoff and Johnson [22], provides an appropriate framework for understanding the cognitive and cultural dimensions of metaphors in human language. The theory posits that metaphors are not merely rhetorical devices but fundamental structures of human thought. Metaphors enable individuals to understand and express abstract concepts, grounding these ideas in basic human perceptions and experiences of the world [23]. Furthermore, conceptual metaphors permeate cognition, language, and behavior, making them essential tools for analyzing meaning-making processes. In this study, the theory is applied to explain the ways in which postgraduate translation students with science and engineering backgrounds interpret and render metaphorical meanings of color words across different text types.

Within the semantic domain of color, metaphors convey cultural, psychological, and cognitive meanings that transcend mere visual perception [24,25]. This highlights the centrality of metaphorical mapping in shaping students’ translation strategies, particularly in contexts where symbolic meanings vary across cultures. According to conceptual metaphor theory, color functions not only as a sensory perception but also as a carrier of metaphorical and psychological meanings embedded in specific cultural contexts. In different cultural frameworks, colors are often used metaphorically to express abstract concepts such as emotions, attitudes, and psychological states. However, postgraduate translation students, particularly those from science and engineering disciplines, may lean toward more literal renderings, emphasizing tangible attributes and overlooking symbolic dimensions [26].

The translation of color words involves both cognitive and psychological mechanisms that shape students’ ability to recognize and convey these metaphorical meanings. Psychological metaphors are especially critical, as they influence whether students can identify and accurately render the emotional or psychological significance of colors in the source language. If these metaphors are recognized and interpreted appropriately, students can select culturally appropriate equivalents that preserve the intended connotations in the target language [23]. Conversely, inadequate recognition of metaphorical meanings may result in translations that are semantically accurate but pragmatically or affectively deficient.

Cultural metaphors also play a vital role in translation. Variations in how colors are culturally interpreted can significantly impact translation strategies. Translators unaware of these nuances may render color words literally, omitting metaphorical meanings crucial to the source text. Conceptual metaphor theory emphasizes that metaphors are intrinsic to cognitive structures. Thus, students’ word choices in translation are not only linguistic decisions but are shaped by their mental representations of cultural and psychological associations [23].

The cognitive mechanisms underlying color translation involve metaphorical mappings and associative thinking. While students may rely on source-language metaphorical frameworks, doing so without adapting to target-language norms may cause misalignment in meaning. For instance, while the color red might symbolize love in some cultures, in others it may represent anger or danger. Failure to decode and re-map such culturally bound psychological metaphors can result in semantically dissonant target texts [24,25].

To navigate these cross-cultural challenges, students are expected to develop both cognitive flexibility and cultural sensitivity, enabling them to recognize and adapt to variations in symbolic and psychological meanings. Effective strategies include annotation, cultural explanation, or adaptation to align the translated color words with the expectations and cognitive frames of the target audience [27]. These strategies not only reflect theoretical principles of conceptual metaphor but also demonstrate how theoretical awareness can inform practical translation decisions.

An innovative aspect of this study was its combination of conceptual metaphor theory and the focus group interview method to explore how students balanced rational analysis with psychological understanding during translation. Conceptual metaphor theory emphasizes how metaphors deeply influence human thought and language structures, with the translation of color words serving as a critical example of metaphorical language. This theoretical framework enabled the study to uncover how students’ translations were shaped by psychological and cultural metaphors. Meanwhile, the focus group interview method fostered interactive discussions that revealed cognitive responses often implicit or overlooked in individual translation tasks. This combined approach provided novel theoretical insights and practical guidance for translation education.

## Methodology

The participants in this study were 21 postgraduate students (12 female, 9 male; aged 23–29) enrolled in master’s programs in Translation and Interpreting at six universities in Shaanxi Province, China. These universities, including Xi’an Jiaotong University, Northwestern Polytechnical University, Xidian University, Xi’an University of Posts and Telecommunications, Shaanxi University of Science and Technology, and Xi’an Technological University, are known for their strong technical profiles in fields such as information technology, science, and engineering. These institutions were deliberately chosen to ensure that the participants had substantial exposure to technical and specialized translation training, which is relevant to the study’s focus on the translation of color words in different text types. Recruitment took place through a postgraduate translation conference at the provincial level, providing a networking platform and conducive environment for participant engagement and the organization of focus group discussions. The recruitment period lasted from March 22, 2025, to April 19, 2025.

To justify data selection and improve representativeness, the discussion prompts were constructed from short and authentic source segments containing color words across three text types (technical, promotional, and literary). Candidate segments were screened using explicit criteria: (1) inclusion of high-frequency basic color terms; (2) coverage of both literal descriptions and metaphorically or culturally loaded uses; and (3) the presence of potential Chinese-English asymmetry in connotation or evaluative meaning. The final prompt set was refined after the first session to ensure comparable difficulty and to elicit a range of strategy choices across genres.

All participants were required to demonstrate strong foreign language proficiency, as evidenced by passing the China Accreditation Test for Translators and Interpreters (CATTI) Band Three, and to have prior experience translating materials from fields such as information technology, electronic engineering, and related areas. Some participants had specific experience in translating technical texts, such as product manuals and scientific documentation. This selection ensured that the cohort was well-positioned to investigate how technically trained postgraduate translation students approach color word translation, particularly how their training influences their ability to navigate both rational analysis and psychological understanding in their decision-making processes.

To investigate how participants translate color words across different contexts, this study employed focus group interviews. The decision to conduct three focus group sessions was based on several methodological considerations. First, dividing the 21 participants into three groups of 6–8 individuals allowed for sufficient diversity within each group while maintaining manageable group sizes. Each group included at least one participant from each of the six universities, ensuring cross-institutional representation and diverse perspectives. This design aimed to maximize representativeness across different technical disciplines, while also fostering a deeper, more varied set of insights. Second, the group size (6–8 participants per session) was selected in accordance with established focus group methodology, which recommends this range for achieving effective group interaction, active participation, and in-depth discussions. This structure helped balance group dynamics, ensuring that each participant had the opportunity to contribute meaningfully to the conversation while preventing dominance by a few individuals. Third, organizing three smaller groups rather than one large group facilitated cross-group interaction. Answers and reflections shared in one group often stimulated discussions in the others, enriching the overall data and enhancing its reliability by enabling comparison and contrast between group perspectives.

Each session lasted between 60 and 90 minutes and followed a semi-structured interview format. The semi-structured design allowed for flexibility in the discussion, while ensuring that key areas of interest were addressed. Before group discussion, participants were first asked to propose an initial translation for each prompt and briefly explain their reasoning, after which alternative renderings and justifications were explored collectively. To prompt reflection and discussion, participants were asked questions such as: “How would you translate the colors in the poetic phrase ‘绿肥红瘦’ (literal translation: green leaves flourish as red flowers wither)?” and “How would you approach translating ‘red’ and ‘green’ in the sentences ‘The laser device emits a warm red beam, clearly marking the target’ and ‘The new design integrates environmental concepts, using eco-friendly green technology’?” These questions encouraged participants to consider visual attributes, as well as cultural and psychological symbolism, and to reflect on the challenges of translating color words that lack direct equivalents across languages and contexts [28,29].

Data collection and analysis were conducted iteratively. Preliminary coding began after the first session, and subsequent interview outlines were refined based on emerging themes. This iterative process helped ensure that the data collection adapted to the flow of discussions and reflected the evolving nature of the inquiry. After the three focus group sessions, no new themes or strategies emerged, indicating thematic saturation and confirming the sufficiency of the data [30]. With participants’ consent, one session was audio-recorded; for the other two sessions, structured note-taking was used to capture key turns and examples, and the notes were expanded immediately after each session into a consistent transcript format. All session records were anonymized and prepared as analytic transcripts for coding.

Qualitative data analysis, including coding and thematic analysis, was used to systematically classify and summarize the data. This process focused on participants’ translation choices, cultural and psychological considerations, and the strategies they employed when dealing with non-equivalent color words [31]. By comparing discussions across the groups, both commonalities and differences were identified, revealing the cognitive and psychological mechanisms underlying students’ translation decision-making processes. The iterative nature of the analysis ensured that the research team was able to capture a comprehensive understanding of how technical translation students navigate the translation of color words.

The study adhered to established ethical standards. All participants provided written informed consent before participation, and ethical approval was obtained from the Ethics Committee of the authors’ affiliated institutions. To protect participants’ identities and ensure the confidentiality of their contributions, pseudonyms (e.g., Student A, Student B, etc.) were used in place of real names. This approach ensured the authenticity of the discussion data while safeguarding participants’ privacy [32].

## Results

### Are students aware of cultural connotations associated with color words?

Most students admitted that they do not consistently reflect on the cultural connotations of color words, particularly in technical texts. For example, Student A noted: *“Sometimes I pay no attention to the cultural connotations of color words, especially when texts are more technical. Colors are just a description, like ‘yellow’ meaning yellow, and ‘red’ meaning red.”* This perception supports Rus and Harpa’s conclusion that technical translations often suppress cultural overtones in favor of clarity [8]. Responses as such indicate that students tend to construe color primarily through what we theorize as the COLOR AS PHYSICAL ATTRIBUTE schema, in which color is cognitively processed as a perceptual and measurable property rather than a symbolic resource.

Nevertheless, Student B acknowledged greater awareness of cultural connotations when encountering literary texts or works with strong cultural significance. He stated,

*If I were translating a classic novel like* Dream of the Red Chamber *(In Chinese,* 红楼梦, *a traditional Chinese literary novel), I would pay more attention to the cultural connotations of colors, such as “red” representing love and passion, while “green” might carry a sense of freshness and immaturity.*

This distinction suggests that students’ awareness of cultural connotations is highly context-dependent. In literary settings, students are more inclined to activate culturally grounded metaphorical mappings, corresponding to the COLOR AS CONCEPTUAL ATTRIBUTE schema (our theorization), in which colors function as carriers of abstract emotion and cultural meaning. Yang’s claim that cultural differences exert greater influence in texts rich in symbolic undertones is thus reflected in this observation [33].

The discussion also revealed students’ awareness of cross-cultural variation in color symbolism. Student C commented: *“Red carries various meanings across cultures: in the West, it often signifies passion or danger; while in China, it usually denotes festivity and good fortune; I therefore adapt my translations accordingly.”* Student D further noted: *“Sometimes, even if I know a color word’s cultural connotations, I feel that worrying too much about misinterpretation can go overboard; being too cautious might distort the original text.”* Such remarks indicate that students are not merely identifying metaphorical meanings but are also evaluating their relevance and communicative cost. This evaluative stance aligns with Puchala’s emphasis on intercultural transfer, where equivalence involves selective adaptation rather than exhaustive cultural explication [15].

Student E illustrated awareness of cultural connotations in translating general words:

*When translating “red”, I usually consider the cultural associations of the color in the target language context. If translating it as “red” makes the reader uncomfortable, I would choose a more psychologically appropriate word like “crimson” or “scarlet”, which better conveys the passion or danger associated with “red” in Western culture.*

These observations suggest that students possess partial but uneven awareness of cultural color metaphors. Their engagement with such metaphors tends to be activated when symbolic meaning is foregrounded by genre or context, rather than being consistently integrated across translation tasks [34]. This pattern reflects a selective activation of the COLOR AS CONCEPTUAL ATTRIBUTE schema, constrained by contextual salience and communicative purpose.

### How do cognitive mechanisms shape their word choices in translation?

The data indicate that cognitive mechanisms play a central role in shaping students’ word choices, particularly when color words lack direct cross-linguistic equivalents. Translation emerges as a process of cognitive negotiation, in which perceptual categorization, associative thinking, and contextual evaluation interact [4,35]. For instance, Student H shared:

*When translating “*蓝天白云*”, I render it as “blue sky and white clouds” because it’s a straightforward description that requires minimal cognitive effort. However, for “*金秋 *(golden autumn)”, I think about the associations autumn evokes: harvest, maturity, and warmth, to convey a richer image.*

This contrast illustrates how students shift between different modes of cognitive processing. While ‘blue sky and white clouds’ is treated under the COLOR AS PHYSICAL ATTRIBUTE schema, ‘golden autumn’ activates metaphorical associations linked to value and maturity, corresponding to the COLOR AS CONCEPTUAL ATTRIBUTE schema.

Some students, however, emphasized a more rational and technical mode of cognition. Student J noted: *“For color words like ‘green’ in technical texts, I focus purely on the visual attributes of the color, without considering its cultural meanings.”* This reflects a functionalist cognitive orientation in which metaphorical mappings are deliberately suppressed to maintain clarity and efficiency [15].

This suppression, however, was not always stable. Student K observed: *“Even in technical texts, ‘green’ can evoke ideas like environmental protection or sustainability, so my mind sometimes automatically considers these associations.”* This comment suggests that certain metaphorical associations, such as GREEN AS ENVIRONMENTALISM, may be cognitively entrenched and activated involuntarily. Such instances demonstrate that metaphorical cognition operates beneath conscious strategy selection and may intrude even in contexts that prioritize literal meaning [27,33].

The role of context-sensitive cognition was further emphasized by Student L: *“For ‘white’, in a technical document, I see it simply as ‘white’. But in art or advertising, it triggers ideas of purity, simplicity, or nobility. My cognition adjusts depending on the context.”* This illustrates how students dynamically regulate metaphorical activation, selecting between perceptual and conceptual construals according to communicative goals [33,35].

Cognitive challenges were particularly evident in cases involving color terms without clear equivalents. Student M explained: *“‘Cyan’ is between blue and green, but there’s no perfect Chinese equivalent. I usually choose ‘*蓝绿色 *(blue-green)’, though sometimes I consider the traditional word ‘*青色 *(greenish blue)’.” She further elaborated: “‘*青色*’ carries strong cultural connotations, whereas ‘*蓝绿色*’ is more neutral. Cognitively, I weigh which associations are relevant to the text before choosing.”* This example illustrates how translators engage in evaluative cognition, balancing perceptual accuracy against metaphorical and cultural resonance [8,33].

Finally, Student N highlighted the cognitive demands of literary translation: *“‘Rose’ can be rendered as ‘玫瑰色 (rose color)’, but in poetry, it carries strong psychological overtones, symbolizing love and romance.”* This observation underscores Stolze’s view that literary translation requires hermeneutic competence, whereby cognitive mechanisms enable translators to reconstruct metaphorical and emotional meaning rather than rely solely on literal equivalence [4].

### How do text types and functions shape their treatment of color words?

Students consistently emphasized that text type and communicative function play a decisive role in determining how color words are treated in translation. Student P explained:

*When translating “yellow”, I am aware that it may signify jealousy or sickness in Western cultures, whereas in China, it’s more commonly associated with happiness and prosperity. However, when translating a technical manual about production processes, I typically disregard these cultural distinctions and translate it simply as “yellow”.*

This response illustrates how technical discourse encourages a construal of color as a physical attribute, reinforcing literal translation and minimizing metaphorical interpretation. Such behavior aligns with a functionalist orientation, where communicative efficiency outweighs symbolic nuance [4,35].

Similarly, Student Q noted that in technical texts she renders “white” simply as “白色”, setting aside its Western cultural associations with purity. This further reflects the dominance of the COLOR AS PHYSICAL ATTRIBUTE schema in contexts where color serves a descriptive or referential function [18].

However, students also recognized that text type does not automatically exclude cultural considerations. Student R described her contrasting approaches:

*If I encounter “blue” in a literary work, I might consider its psychological connotations, such as melancholy or tranquility. In advertising, however, “blue” often conveys professionalism, calmness, or trustworthiness, which influences my translation choices. For example, when translating an advertisement for a high-end brand, “blue” represents the brand’s premium and technological qualities.*

This example demonstrates how the same color term can activate different conceptual metaphors depending on communicative function, such as BLUE AS MELANCHOLY in literature versus BLUE AS TRUST in branding [24,36]. In persuasive genres, some students prioritized immediacy over elaboration. Student S stated: *“In advertisements, if ‘red’ is meant to convey passion and energy, I would simply use ‘red’ without adding annotations.”* This reflects sensitivity to genre constraints, where excessive explanation may weaken affective impact [4,15].

In more culturally loaded genres, however, students saw cultural meaning as integral to communicative adequacy. Student T explained her approach when translating novels about Western aristocracy: *“When translating ‘purple’, I consider its associations with royalty and nobility in Western cultures, whereas, in China, it might be seen as mysterious and dignified.”* For such texts, she sometimes adds explanatory notes (e.g., “In Western culture, purple is often a symbol of royalty and nobility”) to preserve the semiotic function of the original. Yet others, like Student U, cautioned against overloading neutral texts with cultural explanation. He noted:

*Adding annotations for color words might confuse the target readers, especially when cultural differences are not significant. For instance, in Western cultures, brown can symbolize qualities such as earth, reliability, or stability. But if I am translating a furniture catalogue or a technical description of materials, rendering it simply as “*棕色 *(brown)” is sufficient. Chinese readers will understand the intended meaning as a straightforward description of color, and inserting explanations about its symbolic associations would be unnecessary and distracting.*

His view reflects a cost-benefit approach, where symbolic meaning is only invoked if it enhances the communicative purpose of the text [8,15].

### What strategies do they employ for translating color words?

Students’ strategic choices reflect a continuum shaped by context, genre, and perceived metaphorical load. In technical and scientific texts, most students favored literal translation, prioritizing direct correspondence with visual attributes. As Student V explained: *“When translating color words in scientific materials, such as electronic product manuals, I prioritize their direct color attributes, as the meanings are clear and precise and carry no psychological connotations.”* This preference reflects the dominance of the COLOR AS PHYSICAL ATTRIBUTE schema and aligns with prior observations that technical translation privileges objectivity and precision [8].

In contrast, when translating texts imbued with cultural or psychological significance, such as scientific materials, poetry, novels, or film subtitles, students demonstrated a greater inclination to adopt strategies that foreground the symbolic meanings of colors. However, their technical background continued to influence their decision-making. For example, Student V remarked:

*For translating a phrase such as “*绿肥红瘦 *(literally, ‘green leaves flourish as red flowers wither’)”, I would take into account the cultural associations of the colors red and green, particularly in the Chinese context, where “red” is commonly linked to festivity and good fortune, while “green” evokes vitality and nature.*

This suggests that in literary texts, color words often carry complex cultural and psychological layers of meaning. To effectively convey these dimensions, translators may need to consider the cultural background of the target language. As Yang [33] noted, translators need to adapt to the target culture to convey the cultural meanings embedded in the source text with accuracy. However, Student V acknowledged that considering the cultural aspects of a color word is not an automatic process and often requires deliberate reflection. He noted: *“At times, when translating literary works, I may overlook the cultural associations of colors, particularly under time pressure or heavy workload, when my tendency toward technical considerations becomes dominant.”* This suggests that although students acknowledge the importance of cultural associations in translating color words, practical constraints often take precedence over these considerations.

Some students adopted differing strategies. Student W stated:

*In literary translation, I tend to avoid overemphasizing the cultural connotations of colors, focusing instead on preserving the original expression. If I translate “green” and “red”, it might lose the artistic flavor of the original, especially in a poetic sentence like “*绿肥红瘦 *(verdant leaves thrive while blossoms wane, Student C’s translation).”*

This perspective highlights an alternative approach where, beyond cultural considerations, the translator prioritizes preserving the artistic expression of original texts. For Student W, the selection of color words reflects a commitment to faithfully recreating the original’s artistic essence. This free translation approach contrasts with literal translation, emphasizing the beauty and imagery of the poem. This approach aligns with House’s emphasis on textual aesthetics and context sensitivity, reaffirming that translators should consider the poetic function of language [35].

Discussions among students often centered on balancing literal translation and free translation. Student L argued that literal translation is typically the most effective strategy for translating color words in technical texts, where excessive interpretations of cultural meanings are unnecessary. Conversely, Student E contended that literal translation might fail to capture the deeper cultural meanings of colors. Student M shared an example: *“When translating movie subtitles, I realize that color words often convey psychological information, and simple literal translation could lead to misunderstanding.”* Their opposing views mirror what Milostivaya et al. [34] described as the translator’s dilemma: preserving factual precision vs. cultural resonance.

Some students proposed pragmatic strategies to address cultural differences. Student F suggested: *“For color words rooted in distinct cultural traditions, I may supplement the translation with annotations or explanations. For example, when translating ‘red,’ I might provide additional notes to help readers grasp its cultural significance.”* Several students endorsed this approach, arguing that annotations can clarify potential cultural misunderstandings or ambiguities. Stolze similarly emphasized that context-based explanations bridge cultural gaps [4]. Student G provided a specific example: *“For instance, when translating the word ‘black’, I might indicate that in Western cultures, it symbolizes death and sadness, while in Chinese culture, it may connote power or solemnity.”* This approach reflects Puchala’s assertion that text type significantly influences translation strategies, with annotations often being indispensable when addressing cultural differences in color words [15].

However, not all students agreed with the use of annotations. Student R argued: *“Using* *annotations during translation might disrupt the flow of original texts, particularly in literary works, where such additions can interrupt the reader’s experience and distort psychological expression.”* Student K emphasized that the translator’s role is to enable the target audience to understand the source text’s color and psychological connotations without excessive intervention. Instead, he advocated for conveying the symbolic meanings of color words through contextual and cultural implications rather than direct annotations. This perspective underscores the tension between preserving the aesthetic integrity of original texts and explicitly addressing cultural differences, revealing the complexity of translation strategies for color words across diverse contexts.

## Discussion

The findings of this study are best interpreted within the framework of conceptual metaphor theory, which offers a principled account of how color may be construed either as a perceptual entity or as a culturally embedded conceptual resource [22]. The analysis reveals a systematic alternation between two metaphorical schemas theorized in this study: COLOR AS PHYSICAL ATTRIBUTE and COLOR AS CONCEPTUAL ATTRIBUTE. Importantly, this alternation is not idiosyncratic but shaped by genre-specific constraints and communicative priorities.

In technical discourse, color is predominantly processed as an objective, visually grounded property. Translation choices in such contexts reflect the dominance of the COLOR AS PHYSICAL ATTRIBUTE schema, whereby metaphorical projections are intentionally inhibited in favor of perceptual categorization and functional adequacy. From a conceptual metaphor perspective, this inhibition does not indicate a lack of metaphorical competence. Rather, it reflects a context-driven regulation of metaphorical schemas, consistent with the view that multiple metaphorical construals coexist in cognition and are selectively activated depending on situational relevance. This finding refines earlier observations that technical translation minimizes symbolism [8] by identifying the underlying cognitive mechanism responsible for such minimization.

By contrast, literary and promotional texts systematically reactivate metaphorical mappings associated with color. In these genres, color functions as a conceptual vehicle for emotion, value, and cultural meaning, prompting the activation of the COLOR AS CONCEPTUAL ATTRIBUTE schema. Metaphorical associations such as RED AS PASSION, BLUE AS MELANCHOLY, or PURPLE AS NOBILITY become salient interpretive resources guiding translation decisions beyond surface-level equivalence. This pattern confirms that metaphorical salience is genre-sensitive and supports the argument that meaning construction in translation is shaped by culturally entrenched conceptual structures rather than by lexical correspondence alone [33,34].

At the cognitive level, the findings suggest that color word translation involves a process of dynamic metaphor regulation. Translators do not simply apply or ignore metaphorical meanings; instead, they continuously evaluate the relevance and communicative utility of competing conceptual mappings. The treatment of color terms lacking stable cross-linguistic equivalents illustrates this process particularly clearly. Here, translators negotiate between perceptual precision and culturally encoded categorization, revealing how metaphorical cognition operates as an evaluative and selective mechanism rather than a fixed interpretive rule. Translation thus emerges as an adaptive cognitive activity in which metaphorical schemas are activated, attenuated, or neutralized in response to contextual demands.

Text type and communicative function further constrain this regulation. In informational discourse, metaphorical elaboration tends to be minimized, as it may interfere with clarity and instrumental goals [4,35]. In affect-oriented or identity-driven discourse, however, metaphorical activation becomes central to meaning construction. These findings underscore that color words carry variable semiotic weight across genres and that translators’ strategies reflect assessments of communicative payoff rather than uniform norms of fidelity or creativity [15]. In this respect, genre functions not merely as a textual classification but as a cognitive constraint that modulates metaphorical salience.

Theoretically, this study contributes to translation studies by advancing a schema-based account of metaphorical cognition in translation. While previous research has often treated metaphor as an interpretive outcome or analytical label, the present study demonstrates that metaphor functions as a regulatable cognitive schema, whose activation and inhibition are systematically conditioned by discourse context. By distinguishing between COLOR AS PHYSICAL ATTRIBUTE and COLOR AS CONCEPTUAL ATTRIBUTE, the study offers a refined analytical framework for explaining why translation behavior varies across genres. Rather than characterizing such variation as inconsistency or deficiency, the findings highlight a form of adaptive rationality in which translators strategically manage metaphorical schemas to meet differing communicative constraints. This perspective strengthens cognitive approaches to translation by foregrounding schema regulation as a central mechanism underlying translation decision-making.

More broadly, the findings carry implications for cognitive translation research by demonstrating that metaphorical processing in translation is not a binary choice between literal and figurative meaning, but a graded and context-sensitive regulatory process. This perspective encourages future research to move beyond static classifications of metaphor use and to examine how translators dynamically manage competing conceptual schemas under varying communicative constraints. By foregrounding schema regulation as a central explanatory mechanism, the study also offers a theoretical bridge between conceptual metaphor theory and genre-oriented translation research, thereby expanding the analytical scope of cognitive approaches to translation.

Implications and contributions can be summarized in two respects. Theoretically, the findings operationalize conceptual metaphor theory in translation decision-making by showing that translators regulate metaphorical salience in a genre-sensitive manner, rather than simply choosing between ‘literal’ and ‘figurative’ meaning [37]. Practically and pedagogically, the results support a differentiated training routine: students should be taught to diagnose text function first, decide whether a color term primarily serves as a perceptual descriptor or a conceptual-symbolic cue, and then select an appropriate strategy (literal rendering, modulation/creative reformulation, or selective annotation) [38]. In technical translation, this routine helps prevent unnecessary metaphorical projection and protects clarity; in promotional and literary translation, it helps preserve culturally and affectively relevant mappings while maintaining readability.

## Conclusion

This study investigated how 21 Chinese postgraduate students majoring in translation with strong technical profiles translate color words across technical, promotional, and literary contexts. Across the focus-group data, their decisions showed genre-sensitive regulation of metaphorical salience: technical contexts tended to activate COLOR AS PHYSICAL ATTRIBUTE and favor function-oriented literal renderings, whereas promotional and literary contexts more often activated COLOR AS CONCEPTUAL ATTRIBUTE and motivated modulation, creative reformulation, or selective annotation to preserve symbolic meaning. These findings support a schema-based account that links conceptual metaphor theory to translators’ strategic decision-making and suggest that translator education should adopt differentiated training that balances precision with cultural resonance.

This study has inherent limitations. The dataset, derived exclusively from student participants, may not fully capture the nuanced practices of professional translators or teacher-translators who possess more extensive real-world experience. Future research could adopt a comparative design, encompassing students, educators, and practitioners across diverse linguistic and cultural contexts to explore how translation strategies evolve with professional development. Additionally, while the study examines students’ handling of cultural connotations in color words, it does not investigate target-language audiences’ reception and interpretation of translated color metaphors; further work integrating intercultural pragmatics could shed light on cross-cultural variations in meaning perception and enhance the ecological validity of translation strategies.
